# Intracellular galectins sense cytosolically exposed glycans as danger and mediate cellular responses

**DOI:** 10.1186/s12929-021-00713-x

**Published:** 2021-03-04

**Authors:** Ming-Hsiang Hong, I-Chun Weng, Fang-Yen Li, Wei-Han Lin, Fu-Tong Liu

**Affiliations:** Institute of Biomedical Sciences, Academia Sinica, Taipei, Taiwan

**Keywords:** Galectin, Carbohydrates, Glycans, Infection, Autophagy

## Abstract

Galectins are animal lectins that recognize carbohydrates and play important roles in maintaining cellular homeostasis. Recent studies have indicated that under a variety of challenges, intracellular galectins bind to host glycans displayed on damaged endocytic vesicles and accumulate around these damaged organelles. Accumulated galectins then engage cellular proteins and subsequently control cellular responses, such as autophagy. In this review, we have summarized the stimuli that lead to the accumulation of galectins, the molecular mechanisms of galectin accumulation, and galectin-mediated cellular responses, and elaborate on the differential regulatory effects among galectins.

## Background

Galectins are β-galactoside-binding animal lectins that contain at least one carbohydrate recognition domain (CRD) [1]. They can be categorized into three groups: (A) one-CRD-type, which can form homodimers, such as galectin-1; (B) two-CRD tandem repeat-type, which contains two different CRDs in tandem connected by a linker region, such as galectin-4, galectin-8, and galectin-9; and (C) chimera-type galectin-3, which contains a short N-terminal region, a proline- and glycine-rich region, and a CRD in the C-terminal region. Galectins do not contain signal peptides, but can be detected in extracellular space [2]. However, large amounts of galectins are located in the cytosol [3]. Intracellular galectins have been shown to bind intracellular partners and control cell fate in a carbohydrate-independent manner [4]. Carbohydrates on glycoproteins and glycolipids are ligands of galectins. These carbohydrates are present on the cell surface and can be subsequently endocytosed and present on the luminal side of endocytic vesicles. Recent findings have shown that intracellular galectins can also regulate cellular responses in a carbohydrate-dependent manner by binding to glycans displayed on damaged intracellular organelles. Here, we have summarized the contributing stimuli and molecular mechanisms leading to the accumulation of intracellular galectins around damaged vesicles, as well as the subsequent cellular responses and the regulation of these processes.

## Main text

### Intracellular galectins sense versatile stimuli through the recognition of host glycans present on damaged endocytic vesicles

#### Bacterial infection

Bacterial infection triggers galectin accumulation around damaged endocytic vesicles. For example, infection by *Mycobacterium, Shigella, Listeria, Salmonella, Streptococcus, Legionella, Yersinia, Coxiella*, and *Helicobacter* causes damage in phagosomes or lysosomes and triggers the accumulation of different galectins, including galectin-1, galectin-3, galectin-4, galectin-8, and galectin-9 in diverse cell types, such as macrophages, epithelial cells, and microvascular endothelium cells [5–13]. Using immunofluorescence staining techniques, Paz et al. found that galectin-3 accumulated around phagosomes containing bacteria, including *Listeria*, *Shigella*, and *Salmonella*. Using electron microscopy, those authors noted that *Shigella* infection induced galectin-3 accumulation at the limiting membrane of bacteria-containing phagosomes and in tubular structures connected to phagosomes. Those authors also demonstrated that a mutant of galectin-3, which does not bind glycans, did not accumulate around *Shigella*-containing phagosomes, thereby indicating that the lectin activity of galectin-3 is required for its accumulation. Additionally, those authors found that galectin-3 did not accumulate in cells that are deficient in glycans recognized by galectin-3 following *Shigella* infection, which indicated that host glycoconjugates were essential for the assembly of galectin-3-containing structures around *Shigella*-containing phagosomes. Moreover, galectin-3 did not accumulate around *Shigella* deficient in the type three secretion system, thereby indicating that these bacterial components were required for the formation of galectin-3-containing structures [6]. Furthermore, galectin-3 has been shown to directly bind several bacterial components, such as lipid A in lipopolysaccharides (LPS) from *Salmonella* [14, 15]: However, whether these bacterial outer membrane components contribute to cytosolic galectin-3 accumulation around damaged phagosomes that contain bacteria requires further investigation.

Additionally, Thurston et al. observed that *Salmonella* infection induced galectin-3, galectin-8, and galectin-9 accumulation around *Salmonella*-containing vacuoles; however, only galectin-8 contributed to *Salmonella* inhibition. As these galectins failed to accumulate around *Salmonella*-containing vacuoles in cells deficient in mature glycans, they concluded that host glycans mediated the accumulation of these galectins around *Salmonella*-containing vacuoles. Furthermore, they showed that the N-terminal CRD of galectin-8 contributed to its accumulation [8]. Similarly, Pareja et al. reported that infection with *Coxiella* triggered the accumulation of galectins, including galectin-1, galectin-3, galectin-4 galectin-8, and galectin-9. They also observed that galectin-3 accumulated around these phagosomes in the presence of N-acetyl-D-lactosamine, an inhibitor that blocks the interactions between galectin-3 and its ligands at the cell surface, suggesting that accumulated galectin-3 was derived from the cytosol. Furthermore, those authors found that galectin-3 did not accumulate around *Coxiella* deficient in the type four secretion system, which indicated that these bacterial elements were essential for galectin-3 accumulation around *Coxiella*-containing phagosomes. Through the expression of galectin CRD mutants, those authors also observed that the CRD of galectin-3 contributed to its accumulation around *Coxiella*-containing phagosomes. Furthermore, both CRDs of galectin-8 were required for its accumulation around *Coxiella*. Using cells deficient in mature glycans, the authors revealed that host glycans mediated galectin-8 accumulation around *Coxiella*-containing vacuoles. However, whether host glycans or *Coxiella* components are involved in the accumulation of galectin-1, galectin-3, galectin-4, and galectin-9 around *Coxiella*-containing vacuoles remains to be clarified [11].

We also found that *Listeria monocytogenes* infection triggered galectin-3 accumulation around bacteria-containing phagosomes, in which the membrane integrity was compromised by the bacterial pore-forming cytolysin listeriolysin (LLO), as revealed by electron microscopy. We also confirmed that the presence of host complex-type N-linked glycans was required for galectin-3 accumulation. Moreover, galectin-8 was found to accumulate around *Listeria*. However, whether accumulated galectin-8 is involved in *Listeria* replication needs to be further studied [7]. Additionally, we found that *Helicobacter pylori* infection induced galectin-8 accumulation around damaged lysosomes in a human gastric cell line, and less galectin-8 accumulated around damaged lysosomes following *H. pylori* infection when cells were pre-treated with an O-glycosylation inhibitor, indicating that host O-linked glycans were involved in galectin-8 aggregation. As less galectin-8 accumulated around damaged lysosomes following the infection of cells with vacuolating cytotoxin A (VacA)-deficient *H. pylori*, we concluded that VacA of *H. pylori* contributed to lysosomal damage and galectin-8 accumulation. We also observed that lower levels of galectin-8 accumulated in atg5-knockout cells after *H. pylori* infection, which suggested that autophagy enhanced galectin-8 aggregation in *H. pylori*-infected cells. However, the molecular mechanism by which autophagy promotes galectin-8 accumulation needs to be determined [12].

#### Viral infection

Infection with non-enveloped viruses also induces galectin accumulation around damaged endocytic vesicles. For example, using immunofluorescence imaging, Maier et al. found that adenovirus infection triggered the accumulation of galectin-3 around damaged endosomes or lysosomes. Additionally, immunofluorescence imaging at different time points demonstrated that the exposure of adenovirus membrane lytic protein VI (pVI) preceded the recruitment of galectin-3 to endosomal membranes. They also showed that pVI was associated with galectin-3-positive ruptured endosomes, while adenovirus capsids could escape from damaged galectin-3-positive endosomes at later time points [16]. Additionally, Montespan et al. used a similar experimental approach and found that both galectin-3 and galectin-8 accumulated around damaged endosomes containing adenovirus. Furthermore, they found that knockdown of galectin-8 repressed adenovirus transduction, but knockdown of galectin-3 did not have an effect. Moreover, compared with wildtype adenovirus particles, the infection of mutant ts1 adenovirus particles, which fail to release pVI, to human bone osteosarcoma epithelial cells barely triggers the accumulation of galectin-3 and galectin-8, indicating viral factor pVI contributes to endosomal damage and cytosolic galectin accumulation around damaged endosomes. Interestingly, compared with wildtype adenovirus, more galectin-3, galectin-8, autophagy adapter proteins, and autophagy marker proteins accumulated around adenovirus with mutated PPxY motif on viral protein pVI. Furthermore, galectin-8 knockdown, but not galectin-3 knockdown significantly reduced the accumulation of autophagy marker proteins around mutated adenoviruses and promoted the transduction of mutated adenovirus. These results suggest that the PPxY motif on viral protein pVI contributes to adenovirus escape from galectin-positive endosomes and galectin-8-mediated autophagic viral clearance subsequent to virus-induced endosonal damage [17].

Furthermore, using immunofluorescence staining and fluorescence microscopy, Staring et al. observed that infection with picrornaviruses, including poliovirus and coxsackievirus, also induced galectin-8 accumulation. Assessing the expression of truncated galectin-8, those authors demonstrated that the N-terminal domain of galectin-8 contributed to its accumulation around picornavirus-damaged endosomes. Compared with control cells, more viral genomes were located in galectin-8-positive structures in host phospholipase PLA2G16-deficient cells. Additionally, the repression of PLA2G16 inhibited picornavirus infection and increased the survival of viral-infected mice. Therefore, galectin-8 can detect picornavirus-damaged endosomes, but picornavirus utilizes a host factor PLA2G16 to facilitate translocation of its genome away from galectin-8-positive permeated endosomes and escape from galectin-8 directed autophagic clearance [18]. Interestingly, galectins have been reported to bind several viruses directly [19]. Whether the binding of galectin to viral components is also involved in galectin accumulation around damaged endocytic vesicles that contain the virus requires to be further investigated.

#### Pathogenic protein aggregates, mineral crystals, and chemical reagents

Pathogenic protein aggregates also trigger galectin accumulation through causing a rupture of endocytic vesicles. For example, using immunofluorescence staining, several studies have shown that the treatment of intraneuronal protein aggregates α-synuclein and assembled Tau induces the accumulation of galectins, including galectin-3 and galectin-8, in microglia and neuron cells [20–22]. Using light and immunofluorescence microscopy, Maejima et al. reported that monosodium urate crystals and crystalline silica also induce the formation of galectin-3 aggregates around damaged lysosomes [23]. Using similar techniques, Unno et al. found that kidney stone development triggered galectin-3 accumulation around damaged lysosomes in renal tubular cells [24]. As these aggregates have been reported to damage endocytic vesicles and induce cellular or tissue injury, galectin accumulation may be a novel biomarker of cellular or tissue damage in response to various insults. However, few studies have investigated the biological significance of galectin aggregates under these conditions. Siew et al. observed that galectin-3 formed aggregates around damaged lysosomes of Huntington’s disease (HD) microglia. Through the genetic suppression of galectin-3, the authors demonstrated that galectin-3 levels in microglia contributed to HD pathogenesis in a nuclear factor-κB (NFκB)- and nucleotide-binding domain leucine-rich repeat and pyrin domain containing receptor 3 (NLRP-3) inflammasome-dependent manner. Furthermore, experiments using cell-permeable TD139 and cell-impermeable lactose have revealed that intracellular galectin-3 accumulation leads to HD pathogenesis. As galectin-3 knockdown reduced the amount of lysosomal marker LAMP-1 in HD microglia, the authors concluded that galectin-3 accumulation interfered with the clearance of damaged lysosomes [25]. As pathogenic protein aggregates and mineral crystals have been reported to trigger galectin accumulation, it would be of interest to investigate whether galectin aggregates modulate the homeostasis of damaged organelles and contribute to the pathogenesis of these stimuli.

Exposure to chemical reagents also induces galectin accumulation around damaged endosomes or lysosomes. For example, fluorescence and immunofluorescence microscopy showed that treating cells with transfection reagents, including liposomes and calcium phosphate precipitates, triggered the accumulation of galectins, including galectin-3, galectin-4, galectin-8, and galectin-9, around damaged endosomes [26, 27]. However, as galectin-3 directly interacts with membrane phospholipids and cholesterol [28], whether these components on liposomes or glycans from the host cells contribute to galectin accumulation around liposome-damaged endosomes remains to be further identified. Additionally, the treatment of cells with cationic cell-penetrating peptides or polymer-positive nanoparticles was found to induce galectin-8 accumulation [29, 30].

Furthermore, the treatment of cells with light-illuminated photosensitizers, endosome-damaging agents, or lysosome-damaging agents has been shown to induce the accumulation of galectins, including galectin-1, galectin-3, galectin-8, and galectin-9, around damaged endosomes or lysosomes [8, 31]. Electron microscopy revealed that galectin-3 and galectin-8 accumulated around the compromised membrane structure of endosomes damaged by light-illuminated photosensitizers. Moreover, using fluorescence microscopy, we observed the accumulation of galectin-3 around light-illuminated and photosensitizer-containing damaged endosomes when cells were pretreated with cell-impermeable lactose. These results indicate that intracellular galectin-3 contributes to its accumulation around damaged endosomes [31]. As the C-terminal region truncated galectin-3, which is deficient in CRD, failed to accumulate around damaged endosomes, we concluded that the lectin activity of galectin-3 contributed to its accumulation [31]. We also observed that significantly fewer galectins accumulated around damaged endosomes in cells deficient in galectin-recognized glycans, thereby indicating that host carbohydrates were responsible for the accumulation of galectins around illuminated endosomes damaged by photosensitizers. This conclusion was also supported by the observation that galectin-3 accumulation did not occur in the presence of the galectin-3 inhibitor TD139, which targets the CRD of the protein[31].

**Fig. 1:**
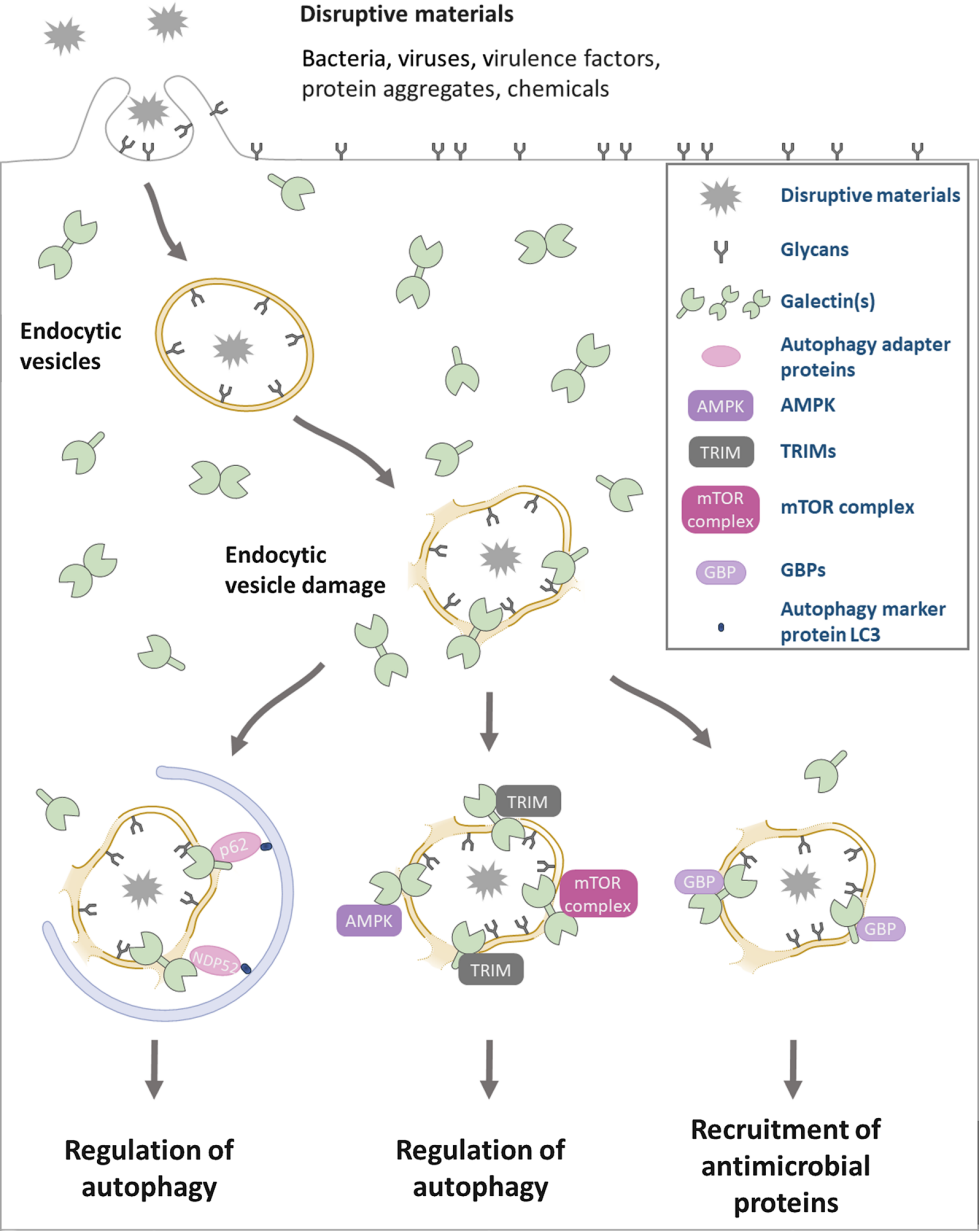
Intracellular galectins recognize cytosolic exposed glycans around damaged endocytic vesicles and control cellular responses, such as the regulation of autophagy and the recruitment of antimicrobial proteins

These findings indicate the mechanism by which galectins sense incoming danger. Initially, pathogens or disruptive materials are endocytosed and confined within the lumen of endocytic vesicles. At later time points, the membrane structures of endocytic vesicles are compromised. Carbohydrates originally present on the cell surface and then displayed on the luminal side of endocytic vesicles become exposed to the cytosol. Cytosolic galectins recognize exposed carbohydrates and accumulate around damaged endocytic vesicles. Finally, accumulated galectins recruit cytosolic binding partners to the foci to regulate cellular responses (see below) (Fig. 1).

### Accumulated galectins recruit cytosolic binding partners or engage cellular protein components around damaged endocytic vesicles

#### Autophagic activation

Thurston et al. observed that galectin-8 accumulates around damaged *Salmonella*-containing vacuoles. The authors also reported that the N-terminal CRD of galectin-8 is responsible for its accumulation, while the C-terminal CRD binds to the autophagy adapter protein NDP52. This was subsequently linked to the recruitment of NDP52 to damaged foci, eventually targeting damaged vacuoles for autophagy and eliminating *Salmonella* [8]. Galectin-3 and galectin-9 were also observed to accumulate around *Salmonella*-containing vacuoles, but they were not involved in *Salmonella* clearance. Thus, the biological significance of galectin-3 and galectin-9 accumulation around *Salmonella*-containing vacuoles remains elusive [8]. Additionally, Cheng et al. demonstrated that *Streptococcus* infection leads to galectin-8 accumulation in *Streptococcus-containing* vacuoles. Studies using galectin-8-deficient cells reported decrease in the level of E3 ligase parkin or ubiquitin-positive *Streptococcus*. Furthermore, they demonstrated that galectin-8 physically interacts with parkin. These results indicate that galectin-8 promotes the accumulation of parkin and ubiquitin to *Streptococcus-*containing vacuoles for the inhibition of *Streptococcus* replication [9].

Chen et al. reported that galectin-3 accumulated around endosomes damaged by calcium phosphate precipitate. Accumulation of the autophagy adapter protein p62, and formation of autophagy marker LC3-positive tubulovesicular structures were suppressed following galectin-3 knockdown under treatment. Therefore, they concluded that galectin-3 promoted the targeting of autophagy to endosomes damaged by calcium phosphate precipitates. The authors also observed that p62 knockdown repressed the formation of LC3-positive tubulovesicular structures induced by calcium phosphate precipitates, thereby indicating that p62 was involved in this process [27]. Chauhan et al. reported that galectins physically interact with TRIM proteins, which are also E3 ubiquitin ligases. Since the knockdown of galectin-3, TRIM16, and ATG16L1 increased the survival of *Mycobacterium*, they also concluded that galectin-3 and TRIM16 cooperated to mediate selective autophagy for protection of cells from *Mycobacterium* invasion [32].

Jia et al. found that both galectin-8 and galectin-9 accumulated around lysosomes in cells exposed to Leu-Leu-O-Me (LLOMe). They discovered that galectin-8 associated with mTOR apparatus on the lysosomes and inhibited mTOR activity, which subsequently promoted autophagy. They also demonstrated that galectin-9 activated AMPK in response to lysosomal injury and consequently led to autophagy [33]. By applying similar experimental strategies, Kimura et al. found that galectin-8 formed aggregates in cells following LLOMe treatment. Galectin-8 interacts with the secretory autophagy cargo receptor TRIM16 and mediates the secretion of cargo IL-1β through secretory autophagy. Galectin-3 was found to accumulate around damaged lysosomes, but did not affect IL-1β secretion [34]. It would be interesting to investigate whether these cellular responses triggered by lysosome-damaging agents also occur in cells exposed to pathogens.

#### Recruitment of antimicrobial factors

Freeley et al. used immunofluorescence staining to show that galectin-3, galectin-8, and galectin-9 accumulated around *Legionella*-containing vacuoles. They also found that galectin-3 directed antimicrobial interferon-inducible guanylate binding proteins (GBPs) to *Legionella-* or *Yersinia*-containing phagosomes. Furthermore, galectin-8 was shown to contribute to the recruitment of GBP to bacteria-containing phagosomes. Mutant *Legionella* and *Yersinia* strains revealed that the type four secretion system of *Legionella* and the type three secretion system of *Yersinia* promoted the concomitant recruitment of galectin-3 and GBPs to pathogen-containing vacuoles. The authors concluded that vacuolar damage mediated by the bacterial secretion apparatuses provided a molecular pattern of pathogenesis, which triggered the targeting of GBPs through galectins [10]. Interestingly, the bacterial loads were similar in wildtype and galectin-3-deficient bone marrow-derived macrophages after bacterial infection, which suggested that other factors, such as galectin-8-mediated GBP recruitment or galectin-8-mediated autophagic clearance, might also be involved in bacterial inhibition.

### Differential accumulation patterns and consequences of accumulation among galectins

#### Galectins differentially accumulate around and dissociate from damaged vesicles

Pareja et al. found that infection with *Coxiella* induces the accumulation of galectins, including galectin-1, galectin-3, galectin-4, galectin-8, and galectin-9, to *Coxiella* replicative vacuoles. They also observed that different galectins associated with and dissociated from *Coxiella* replicative vacuoles (CRVs) at different time points [11]. Similarly, via time-lapse confocal microscopy, galectin-8 was found to immediately accumulate around damaged endosomes containing photosensitizers after light illumination, while the galectin-3 accumulation was gradual [31]. These findings suggest that galectins may differentially respond to vesicular damage and play distinct roles during pathogen infection. Conversely, Thurston et al. reported that while galectin-3, galectin-8, and galectin-9 accumulated around endosomes damaged by hypertonic medium and lysosomes damaged by glycyl-L-phenylalanine 2-naphthylamide (GPN), galectin-1 only accumulated around lysosomes damaged by GPN [8]. Collectively, these findings suggest that galectins display differential spatiotemporal characteristics in response to vesicle damage. However, the underlying mechanism and biological significance of differential accumulation patterns among galectins have not been determined.

Using electron microscopy and immunofluorescence staining, we observed the accumulation of both galectin-3 and galectin-8 around damaged endosomes in cells that endocytosed photosensitizers and subsequently exposed to red light. Intriguingly, using super-resolution microscopy imaging, we found that galectin-3 and galectin-8 localized at different microdomains around damaged endosomes. This implies that different galectins may localize at different regions around damaged vesicles to recruit distinct adapter proteins and play varying roles in coordinating cellular responses. Nevertheless, the molecular mechanisms underlying the differential distribution of galectin-3 and galectin-8 around damaged vesicles remain to be determined [31].

#### Galectin-3 accumulation either promotes or inhibits autophagy under different stimuli

Notably, galectin-3 accumulation differentially modulates cellular autophagy when cells are subjected to different challenges. For example, using immunofluorescence staining, we showed that accumulated galectin-3 promoted the autophagy of light-damaged endosomes in galectin-3-EGFP-expressing and galectin-8-deficient cells [31]. However, Cheng et al. reported that accumulated galectin-3 inhibited galectin-8-mediated ubiquitination of *Streptococcus* and promoted *Streptococcus* replication [9]. Similarly, galectin-3 promotes autophagy in a p62-dependent manner when cells are challenged with disruptive chemicals, such as calcium phosphate precipitates [27]. However, our research group found that galectin-3 inhibited autophagy following the infection of cells with *Listeria*. This can occur in the absence of galectin-8, suggesting that galectin-3 inhibits autophagy not through competing with galectin-8 in binding to host glycans on damaged phagosomes [7]. Nevertheless, the factors contributing to differential galectin-3-mediated cellular responses under diverse stimuli require further identification. Furthermore, the molecular mechanisms underlying the contribution of accumulated galectin-3 to autophagic inhibition in *Listeria* infection remain to be determinated.

### Regulation of galectin accumulation and galectin‐mediated autophagy

#### Effect of galectin expression during infection

Cheng et al. found that galectin-8 contributed to parkin-mediated ubiquitination of *Streptococcus* and the autophagic activation that inhibited *Streptococcus* replication in human lung carcinoma epithelial A549 cells, which expressed relatively low levels of galectin-3. The authors found that overexpression of galectin-3 in A549 cells diminished the accumulation of galectin-8, parkin, and ubiquitin around *Streptococcus*, which ultimately promoted *Streptococcus* replication. Conversely, repression of galectin-3 expression in human microvascular endothelial cell line-1 (HMEC-1) cells, which normally express relatively high levels of galectin-3, promotes the galectin-8-mediated ubiquitination of *Streptococcus* [9]. These findings suggest that differential levels of galectin expression regulate galectin-mediated cellular responses, which may compete during pathogen infection.

#### Effect of alterations in carbohydrate composition

We have previously demonstrated the contribution of carbohydrates to galectin-3 and galectin-8 accumulation around vesicles damaged by light-illuminated photosensitizers [31]. We also demonstrated that the removal of cell-surface sialic acids reduced galectin-8 accumulation around damaged vesicles and autophagy, while augmented galectin-3 accumulation as well as autophagic response toward the damaged vesicles. Overall, our findings showed that galectin-3 and galectin-8 played similar and compensatory roles in response to carbohydrate changes following endosomal damage due to light-illuminated photosensitizers [31]. Similar molecular patterns of galectin accumulation that commensurate with carbohydrate alterations can also be observed during *Listeria* infection. We found that galectin-3 accumulated around *Listeria-*containing vacuoles; however, in contrast to endosomal damage induced by illumination, galectin-3 inhibited autophagy and therefore promoted *Listeria* replication. The removal of sialic acids enhanced galectin-3 accumulation around *Listeria-*containing vacuoles, which further inhibited the antibacterial autophagic response, and subsequently promoted *Listeria* replication [7]. Bacteria produce various glycosidases known to be capable of digesting host glycoconjugates including cell surface glycans [35, 36]. It is conceivable these bacteria can modify host glycans displayed on damaged endocytic vesicles and regulate galectin-mediated cellular responses. Taken together, our findings support the concept that intracellular galectins sense changes in cell-surface carbohydrates in the host, which are commonly observed during pathogen infection, inflammation, and tumor progression, and subsequently control cellular responses.

## Conclusions

Intracellular galectins recognize host glycans on damaged endocytic vesicles that become exposed to the cytosol, including damaged phagosomes, endosomes, and lysosomes in response to various stimuli, around which they form aggregates. Accumulated galectins modulate cellular responses that commensurate with the cell-surface carbohydrate composition. This suggests that intracellular galectins are central cellular modulators, which sense incoming danger and environmental fluctuations, transmit them into the cells, and initiate corresponding cellular responses. Nevertheless, several questions need to be addressed. For example, galectin-3, galectin-4, and galectin-8 recognize blood group B antigens expressed on the side chains of bacterial surface LPS of blood group positive bacteria, and the binding of galectin-4 and galectin-8 to bacteria disrupts membrane integrity and kills the bacteria [37, 38]. It would be interesting to determine whether intracellular galectins directly recognize bacterial carbohydrate determinants and initiate cellular responses against phagocytosed bacteria. Furthermore, we found that galectin-3 and galectin-8 were distributed in different microdomains around *Salmonella*-containing phagosomes (data not shown). As the N-terminal region of galectin-3 binds to *Salmonella* LPS [15], further research is needed to determine whether galectin-3 binds to and protects bacteria from autophagic clearance.

Galectins have variable affinity for various glycans on cell surfaces. For example, LacNAc structures of complex N-linked glycans are the major ligands of galectin-3 and the C-terminal domain of galectin-8 [39], and these structures are the major ligands for mediating cytosolic accumulation of galectin-3 and galectin-8 in Chinese Hamster Ovary (CHO) cells [31]. Interestingly, sialic acids on O-linked glycans or glycolipids are ligands of the N-terminal domain of galectin-8 in CHO cells [39, 40]. We observed that galectin-8 still accumulated in CHO-Lec1 cells, which did not express LacNAc structures on complex N-linked glycans but express sialic acids on O-linked glycans or glycolipids [31]. However, galectin-8 did not accumulate around damaged endosomes in CHO-Lec3.2.8.1 cells, which do not express LacNAc structures on complex N-linked glycans and express a much lower level of sialic acids compared with CHO-Lec1 cells [31, 41]. These results suggest that sialic acids on O-linked glycans or glycolipids are ligands for cytosolic galectin-8 accumulation in the absence of LacNAc structures on complex N-linked glycans. Collectively, as the expression levels of galectins are different in cells from different origins, we conclude that the repertoire of the galectin level and carbohydrate composition controls the accumulation of cytosolic galectins around damaged endocytic vesicles and cellular responses.

Infection with *Listeria* and *Streptococcus* induces galectin-3 accumulation and inhibits selective autophagy for bacterial clearance, suggesting that different pathogens hijack intracellular galectin-3 during infection and modulate cellular autophagy for replication. We also found that inhibition of galectin-3 expression repressed bacterial replication [7, 9]. It would be of interest to determine whether pharmaceutical inhibition of intracellular galectin-3 to block the recognition of carbohydrates by galectin-3 will control bacterial replication. We found that TD139, an inhibitor that blocks the binding of galectin-3 to its carbohydrate ligands, could penetrate the cells and reduce galectin-3 accumulation around damaged vesicles [31]. It may be worthwhile to develop specific galectin inhibitors as potential and novel therapeutic strategies to combat pathogen-associated infections.

Infection with non-enveloped viruses triggers the accumulation of galectins around damaged endosomes, and viruses have evolved strategies to utilize viral components or host factors to escape galectin-positive endosomes [17, 18]. However, it would be of interest to determine whether these galectin-positive structures contribute to virus replication. In contrast to bacterial inhibition, the authors showed that galectin-8 knockdown suppressed adenovirus transduction, suggesting that galectin-8 promoted adenovirus replication [17]. Furthermore, adenovirus infection was also found to induce autophagy. Moreover, through the knockdown of Atg5, the requirement of autophagy for the efficient nuclear transport of adenovirus was observed [17]. As galectin-8 has been reported to accumulate around damaged endosomes and target them for autophagy, these findings suggest that galectin-8-positive structures may indirectly promote adenovirus replication through a similar process. Furthermore, it has also been reported that autophagy promotes picornavirus replication [42]. As picornavirus infection induces galectin-8 accumulation around damaged endosomes, it would be of interest to investigate whether galectin accumulation regulates cellular responses and subsequently contributes to picornavirus replication.

In addition to the stimuli listed above, additional insults have been reported to induce the accumulation of galectins, including oxidative stress and anticancer drugs [43, 44]. However, it is not fully understood whether galectin accumulation in response to these challenges regulates cellular responses and cell fate. Similarly, liposomes and specialized nanoparticles, which are commonly used in the delivery of biomaterials, lead to galectin accumulation [26, 29, 30]. Whether these treatments trigger galectin-mediated cellular responses and subsequently contribute to tissue damage should be further investigated. It would be worthwhile to comprehensively study the functions of intracellular galectins in various research areas in order to precisely develop strategies that modulate cellular responses.

## Data Availability

Not applicable.

## Abbreviations

CRD: Carbohydrate recognition domain
LPS: Lipopolysaccharides
LLO: Listeriolysin
VacA: Vacuolating cytotoxin A
pVI: Lytic protein VI
HD: Huntington’s disease
NF-κB: Nuclear factor-κB
NLRP-3: Nucleotide-binding domain leucine-rich repeat and pyrin domain containing receptor 3
LAMP‐1: Lysosomal-associated membrane protein 1
NDP52: Nuclear domain 10 protein 52
p62: Ubiquitin-binding protein p62
LC3: Microtubule-associated protein 1A/1B-light chain 3
TRIM: Tripartite motif containing proteins
ATG16L1: Autophagy related 16 Like 1
LLOMe: Leu‐Leu‐O‐Me
mTOR: Mammalian target of rapamycin
AMPK: AMP-activated protein kinase
IL-1β: Interleukin 1β
GBPs: Interferon‐inducible guanylate binding proteins
GPN: Glycyl‐Lphenylalanine 2‐naphthylamide
